# Brief Research Report: How Do Claw Disorders Affect Activity, Body Weight, and Milk Yield of Multiparous Holstein Dairy Cows?

**DOI:** 10.3389/fvets.2022.824371

**Published:** 2022-02-25

**Authors:** Luisa Magrin, Giulio Cozzi, Isabella Lora, Paola Prevedello, Flaviana Gottardo

**Affiliations:** Department of Animal Medicine, Production and Health, University of Padova, Padova, Italy

**Keywords:** dairy cow, lameness, automatic milking system, behavior, rumination, milk yield

## Abstract

Claw disorders are among the most relevant health problems in dairy herds. Despite being often not clearly visible and not easily detectable for farmers, they may appear as peculiar cow behavioral and performance patterns. This retrospective study aimed to assess cow's behavior and production variations associated with claw disorders. The study involved 54 lactating Italian Holstein cows reared on the same dairy farm. A veterinarian performed the routine hoof trimming every 6 months, diagnosing specific claw disorders. Multiparous cows with no disorders at the first trimming were selected and monitored for the two following trimming sessions. Data coming from the automatic milking system and neck collars and related to the 15 days before a given cow was diagnosed with claw problems during trimming were further collected. These data were compared with those recorded for the same animal over the 15 days preceding the previous trimming in which no claw disorders were observed. Compared to when they had no disorders, the cows affected by claw disorders had a lower daily activity (405 vs. 429 ± 27.7 units/day, *p* < 0.001), showing a constant decrease in the last 10 days before the trimming, a lower milk yield (26.5 vs. 28.4 ± 1.57 kg/day, *p* = 0.03), and only a decreasing trend of rumination time. These patterns of activity, milk yield, and rumination characterizing cows affected by claw disorders should promote the development of specific algorithms that would enable early detection of lameness thanks to the deviations of these parameters that are sensitive to cow claw health.

## Introduction

In recent years, lameness and claw disorders have continued to be identified worldwide as severe problems in dairy herds due to their negative impacts on cow welfare and farm economy (1, 2). However, farmers tend to underestimate the prevalence of lameness of their dairy herds and perceive a prompt treatment of moderately lame cows as not being so urgent (3). Mild changes in gait and limb movements, lying duration, or eating and rumination times that are possibly caused by claw disorders are not clearly visible, and therefore they are not easy for farmers to detect, they require specific devices that could warn them about the onset of a lameness event (4–6). Several researchers identified interesting associations between lameness events and changes of dairy cows' behavior using diverse automatic lameness detection technologies, both in conventional herds and in those equipped with automatic milking systems (AMS) (1, 6–8). The widespread use of AMS and associated sensor systems such as collars, pedometers, and accelerometers to assess cow's heat and activity might allow the routine collection of specific lameness-induced behavioral changes or performance losses. In this regard, this retrospective study that used productive and behavior data downloaded from storage memories of AMS and neck collars aimed at identifying variations in activity, rumination time, body weight, milk yield, and milking behavior of multiparous Holstein cows over the 2 weeks that preceded the diagnosis of specific claw disorders during the routinely scheduled herd's hoof trimming.

## Methods

### Animals and Farm Management

In this study, data were collected over 2 years (2018–2019) on a commercial dairy farm located in the Po Valley, in the province of Vicenza, North-Eastern Italy. The dairy herd consisted of 54 lactating Italian Holstein cows, housed in a free-stall barn with straw-bedded cubicles. Resting areas were equipped with forced ventilation systems, and traffic alleys were made of a grooved concrete floor automatically cleaned by a scraper once a day. All cows were milked by an AMS (Lely Industries N.V., Maasland, NL), and during the study, they were fed a partial mixed ration that was provided once a day and that they could access *ad libitum*. The composition of the ration was as follows: 50% cereal meals, 5% soybean-based commercial protein mixtures, and 45% mix of hay and straw. This ration was supplemented together with 4 kg per day, on average, of pelleted concentrate offered to each cow during its daily visits to the AMS.

### Routine Functional Hoof Trimming

According to the farm management practices, every 6 months, the same veterinarian routinely inspected and trimmed front and hind hooves of all lactating cows. After the trimming of each animal, the veterinarian recorded the presence/absence of specific disorders on its hooves. The recognition of the disorders was consistent with the protocol proposed by the ICAR claw health atlas international organization (9). Namely, the claw disorders diagnosed by the veterinarian were digital dermatitis (DD) and claw-horn disruption lesions (CHDL), such as sole hemorrhage, white line abscess, and sole ulcer (10). All disorders were recorded as binary measures (presence/absence).

### Behavioral Parameters, Milk Yield, and Body Weight

Cows' activity and rumination time were measured and recorded by using neck collars (SCR Engineers Ltd., Netanya, IL, USA). The collars contained an accelerometer, a microphone, and a microprocessor to identify, record, and summarize cow's activity and rumination sounds every 2 h. A logger included in the collars permitted the storage of data. Cow's activity was expressed as units per cow per day, a unit-less measure that included both steps and neck movements during walking and mounting (1). Ruminating time was defined as minutes of rumination per cow per day. Milk yield, number of daily visits to the AMS, and body weight were recorded by the AMS daily. Information about parity, days in milk, and mature equivalent milk production (MEP) was also extracted for each cow from the AMS management software.

### Experimental Subset of Cows and Data Recording

The study focused only on multiparous cows and, in particular, considered animals that had no claw disorders at the first trimming session, which took place in April 2018. This selection criterion led to a subset of 22 lactating cows that were monitored over the two following trimming sessions, in November 2018 and May 2019. To assess the impact of claw disorders on cows' behavior and performances, data regarding milk yield, body weight, rumination, and activity were downloaded from AMS and collars memories at a later time than that of the three hoof trimming events. In particular, the download considered data recorded over the 15 days preceding the trimming when a given cow was diagnosed with claw problems that were compared with those recorded for the same animal over the 15 days preceding the previous trimming event in which no claw disorders were observed.

### Statistical Analysis

All data were averaged per cow per day, and the single animal was used as experimental unit for all the considered variables. Data of activity, rumination time, body weight, milk yield, and milking frequency recorded over the 15 days before the first trimming in which all the 22 cows were diagnosed without any claw disorders were analyzed with a statistical model that considered only the fixed effect of the day before trimming. For cows diagnosed without claw disorders in a given trimming event but affected by them in the subsequent one, data regarding activity, rumination time, body weight, milking frequency, and milk yield recorded during the 15 days before these two trimming sessions were analyzed with a mixed model that considered the fixed effects of cow's claw health (no disorders vs. presence of claw disorders), day before trimming, and their interaction. Both models included the repeated effects of cow and day before trimming, and the Bonferroni adjustment option. Individual days in milk was included as a covariate to reduce the possible bias due to the different stages of lactation of the cows. The 305-day MEP of the lactation preceding the onset of the study was also included in the models as a covariate to minimize the effect of the different genetic merit of cows. All data were processed by using SAS 9.3 (S.A.S. Institute Inc., Cary, NC, USA). The minimum threshold of statistical significance was set at *p* < 0.05.

## Results

**Figure 1:**
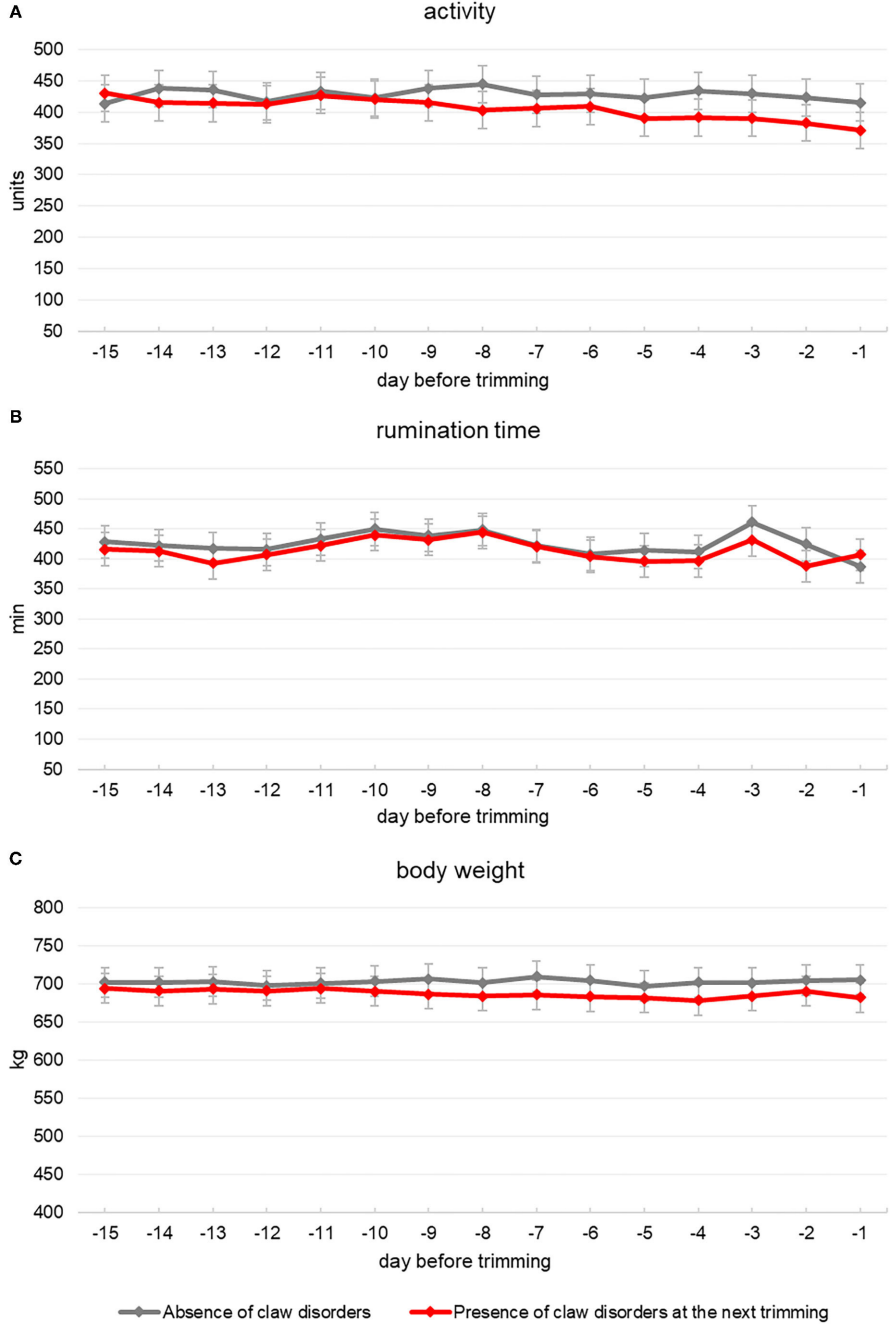
Daily activity **(A)**, rumination time **(B)**, and body weight **(C)** patterns of 15 Italian Holstein multiparous cows over the 15 days that preceded two following hoof trimming practices in which they were diagnosed with no claw disorders (gray line) or affected by claw disorders (red line), respectively.

**Figure 2:**
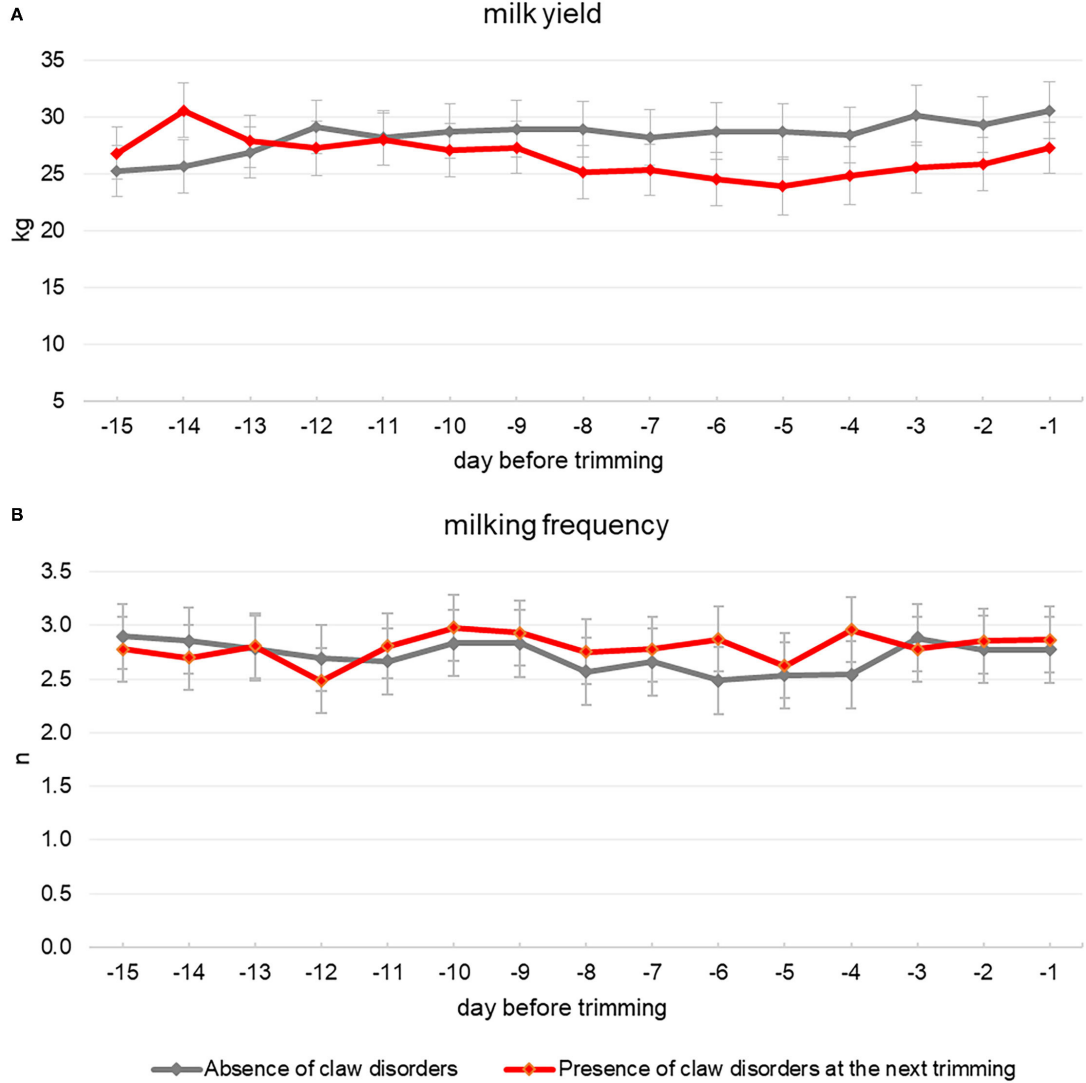
Daily milk yield **(A)** and milking frequency **(B)** patterns of 15 Italian Holstein multiparous cows over the 15 days that preceded two following hoof trimming practices in which they were diagnosed with no claw disorders (gray line) or affected by claw disorders (red line), respectively.

In this retrospective study, the behavior and productive performances of 22 multiparous Holstein cows that were diagnosed without claw disorders by the veterinarian during a first hoof trimming session carried out at 216 ± 119 days in milk (mean ± SD) were further downloaded from AMS and collars. Descriptive statistics about cows' activity, rumination, body weight, and milk production recorded during the 15 days before this trimming session are reported in Table 1. Two following sessions were carried out approximately 6 and 12 months later, respectively. For 7 out of 22 cows, no claw disorders were diagnosed during these next two trimming sessions. Meanwhile, during the second and third sessions, 9 and 6 cows, respectively, were subsequently diagnosed with some claw disorders. On average, claw disorders were recorded on cows that were at 179 ± 83.0 days of lactation. The same animals had been diagnosed without any claw problem about 6 months earlier when they were at 199 ± 122 days of lactation. Among cows with claw disorders, 10 animals were diagnosed with CHDL and 5 with DD. Cows affected by claw disorders showed a lower daily activity if compared to their previous trimming when they had no claw disorders (Table 2). As regards cows' daily activity, a significant interaction seemed to appear between claw health factor and days before trimming (Table 2), and the plot of the least-squares means for the two levels of claw health factor showed a constant decrease in the activity for cows affected by disorders in the last 10 days preceding the trimming event (Figure 1A). Cows affected by claw disorders tended to spend less time ruminating compared to when they had no disorders (Table 2). Daily rumination time was different over the 15 days before trimming, but there was no significant interaction between claw health and day from trimming (Table 2 and Figure 1B). Body weight of cows was affected by claw health (Table 2), as it was lower for affected cows compared with their previous trimming event where they had no disorders. Body weight did not change according to the day before trimming, and no significant interaction between claw health and day from trimming was observed (Table 2 and Figure 1C). Claw health significantly affected cows' milk yield and no significant interaction between claw health and day from trimming was observed for this parameter (Table 2). During the 15 days preceding the trimming, the daily milk yield pattern showed a constant and progressive reduction when cows were affected by a claw disorder compared to the previous trimming session when the same animals had no disorders (Figure 2A). No significant effect of claw health was observed for the average number of daily visits to the AMS or for its interaction with day from trimming (Table 2). This parameter showed a great variability along all the 15 days preceding the trimming events (Figure 2B).

**Table 1:** Daily activity, rumination time, body weight, and milk yield of 22 multiparous Italian Holstein cows during the 15-day period preceding a first hoof trimming session in which they were diagnosed without claw disorders.

		**Mean**	**SD**	**Day before trimming (*p*-value)**
Activity	units/day	388	98.1	<0.001
Rumination time	min/day	424	92.1	<0.001
Body weight	kg/day	696	55.3	0.003
Milk yield	kg/day	32.0	10.5	0.002
Milking frequency	*n*/day	2.72	0.69	0.287

**Table 2:** Effects of claw health (absence of disorders, presence of disorders) on daily activity, rumination time, body weight, milk yield, and milking frequency of 15 multiparous Holstein cows during the 15-day period preceding two hoof trimming events, at the former of which they were diagnosed without claw disorders, and at the latter of which they were found affected by some disorders.

	**Unit/day**	**Claw health**		**SEM**	***p*** **-value**		
		**Absence of disorders**	**Presence of disorders**		**Claw health (CH)**	**Day before trimming (DTrim)**	**CH×DTrim**
Activity^*^	units	429	405	27.7	<0.001	0.01	0.07
Rumination time^*^	min	426	414	22.4	0.08	0.005	0.98
Body weight^*^	kg	703	688	18.3	<0.001	0.99	0.99
Milk yield^*^	kg	28.4	26.5	1.57	0.03	0.97	0.39
Milking frequency^*^	*n*	2.72	2.80	0.27	0.23	0.64	0.81

* *Significant covariate effect of days in milk (p < 0.001) and non-significant covariate effect of MEP (p > 0.05)*.

## Discussion

Regular hoof trimming is the most common preventive practice against cow's lameness since it helps detect and diagnose specific claw disorders and treat them promptly, thus reducing the severity of the clinical cases and the occurrence of new claw diseases (2). This retrospective study aimed at evaluating the effects of claw disorders on production and behavior of cows through the download and processing of data collected in the memories of AMS and collars at a time later than the three trimming events. Therefore, no direct-behavioral observations in the days preceding the three trimmings were available, and cows were considered affected or not by claw disorders according to the vet report at every trimming. Activity and rumination of the 22 selected multiparous Italian Holstein cows at the first hoof trimming when all animals were diagnosed without claw disorders showed some variability for these behavioral parameters. This variability should be considered plausible as any cow has its own behavioral pattern, which depends on its character firstly, and which can be influenced either by physiological changes (i.e., around estrus events) or by the responses to environmental stressors (i.e., changes in THI or feeding, etc.) (11–13). The 15 multiparous Holstein cows diagnosed with claw disorders during one of the following trimmings showed a prevalence of CHDL of almost 67%. It is probable that the high genetic merit of these animals, which produced approximately 30 kg of milk per day, made them more vulnerable to these metabolic-mechanical disorders. Indeed, several authors (2, 14) suggested that high-producing dairy cows are more likely to develop lameness. Regarding cow's behavior, in the current study, it is noteworthy that cows affected by claw disorders were less physically active, especially during the last days before their diagnosis of hoof problems and, according to the literature (1, 15–17), they probably spent a longer time lying down. Several studies have reported a relevant reduction in daily and overnight activity of lame cows compared with non-lame ones in both conventional (18) and AMS herds (1). Also for moderately lame animals, Weigele et al. (17) found reduced average locomotor and neck activities. The impact of lameness on rumination time is still poorly understood and often considered not so significant (17, 19). Some researchers suggested that ruminative behavior can change according to the type of disorder, with cows affected by DD or sole ulcers spending more time ruminating while standing than healthy ones (19). Although this finding cannot be demonstrated in the current study, as the recording system did not record the posture the cows had while ruminating, it was possible to observe a tendency for some impairment of rumination time for cows affected by claw disorders. The fact that the affected cows spent less time ruminating might be due to a reduction in their feed intake as a result of either less time spent eating (20–22) or less feeding bouts (23). This assumption could also be supported by the reduction of body weight that cows showed when affected by a claw disorder. Low body weight was widely reported to be a risk factor for lameness (24), however, we cannot exclude that the lower body weight recorded for lame cows was also related to their different stage of lactation. Recent studies conducted in AMS herds reported a drop in milk yield for lame cows (1, 8, 16, 25). Consistently with this finding, also in this study, lame cows had a lower milk yield compared to when they had no claw disorders. Claw disorders could have impaired cows' feed intake, thus limiting the nutrient supply available for milk production.

Despite the limited number of cows involved in this study, it was shown how some variations in behavior and production of multiparous cows can be associated with claw disorders. The patterns recorded over the 15-day observation period before trimming show that parameters such as activity, body weight, and milk yield are sensitive to cow's claw health as they significantly decreased for affected cows compared with their previous trimming event where they had no disorders. Therefore, the widespread use on dairy farms of automatic devices/sensors, which allow the continuous recording and storage of individual cow data, would enable the early identification of lame cows thanks to anomalous deviations from their usual behavioral and productive parameters. A prompt treatment of these animals could prevent more severe claw problems, and benefit farm profits by reducing herd's milk loss and culling rate.

## Data Availability Statement

The raw data supporting the conclusions of this article will be made available by the authors, without undue reservation.

## Ethics Statement

Ethical review and approval was not required for the animal study because all procedures on cows *in vivo* were carried out by the farm veterinarian within the regular on-farm practices. None of the animals was submitted to painful procedures or mutilations, nor used specifically for scientific purposes requiring the approval of the ethical board.

## Author Contributions

FG and GC conceived and designed the study. LM and PP collected, complied, and analyzed the data. LM, IL, and GC drafted and edited the manuscript. All authors contributed to the article and approved the submitted version.

## Funding

This study was financially supported by the Veneto Region through the Rural Development Program 2014-2020, Action 16.1, EIP-AGRI Operational Groups STALLA 4.0 (DGR Veneto n. 2175, 23/12/2016).

## Conflict of Interest

The authors declare that the research was conducted in the absence of any commercial or financial relationships that could be construed as a potential conflict of interest.

## Publisher's Note

All claims expressed in this article are solely those of the authors and do not necessarily represent those of their affiliated organizations, or those of the publisher, the editors and the reviewers. Any product that may be evaluated in this article, or claim that may be made by its manufacturer, is not guaranteed or endorsed by the publisher.
